# Refractory hemorrhagic esophageal ulcers by Candida esophagitis with advanced systemic sclerosis

**DOI:** 10.1002/jgh3.12353

**Published:** 2020-05-12

**Authors:** Kazuki Natsui, Atsunori Tsuchiya, Shuji Terai

**Affiliations:** ^1^ Division of Gastroenterology and Hepatology Graduate School of Medical and Dental Sciences, Niigata University Niigata Japan

## Abstract

A 64‐year‐old woman diagnosed with rheumatoid arthritis (RA) and systemic sclerosis (SSc) was admitted to our hospital with chief complaints of uncontrolled bleeding from esophageal ulcers and an inability to consume meals. For RA and SSc, she was treated with prednisolone and abatacept and was taking vonoprazan as prophylaxis for steroid‐induced gastric ulcers. She was diagnosed with severe Candida esophagitis, with multiple large and small ulcers with bleeding, based on esophagogastroduodenoscopy and pathological findings. We performed comprehensive treatment; abatacept was discontinued, and total parenteral nutrition was initiated along with antifungal therapy. Improvement in the esophageal ulcers was observed. Although severe Candida esophagitis is a rare condition, we should keep in mind that severe Candida esophagitis can occur in patients with an immunosuppressive compromised host and esophageal movement disorders such as SSc. Regular follow up by endoscopy and prophylactic treatment to prevent severe esophagitis may be necessary.

## Graphical abstract

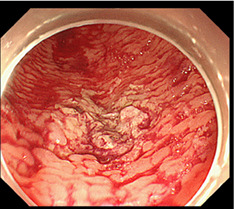

We experienced a rare case of bleeding esophageal ulcers caused by Candida in a patient of systemic sclerosis with immunosuppressive agents. Comprehensive treatment successfully treated these ulcers.

## Introduction

Candida esophagitis is the most common type of infectious esophagitis. Normally, *Candida* is a symbiont of the esophagus and rarely causes critical clinical symptoms. Systemic sclerosis (SSc) is characterized by vasculopathy, fibrosis, and inflammation and mostly affects small vessels and connective tissue.^1^ Gastrointestinal tract involvement is very common, and the esophagus is the most frequently affected part, seen in up to 90% of patients.^2^, ^3^, ^4^, ^5^ Usually, 0.3% and 5.2% of Candida esophagitis cases in the healthy population are detected endoscopically and pathologically, respectively.^6^ However, in patients with an underlying disease, the usage of immunosuppressive agents, acid suppression therapy, malnutrition, diabetes mellitus, chemotherapy, radiation therapy, malignancy‐bearing condition, and smoking were representative causes of Candida esophagitis, and multiple causes were related simultaneously.

We report a rare case of refractory hemorrhagic esophageal ulcers due to Candida esophagitis with advanced SSc.

## Case Report

**Figure 1:**
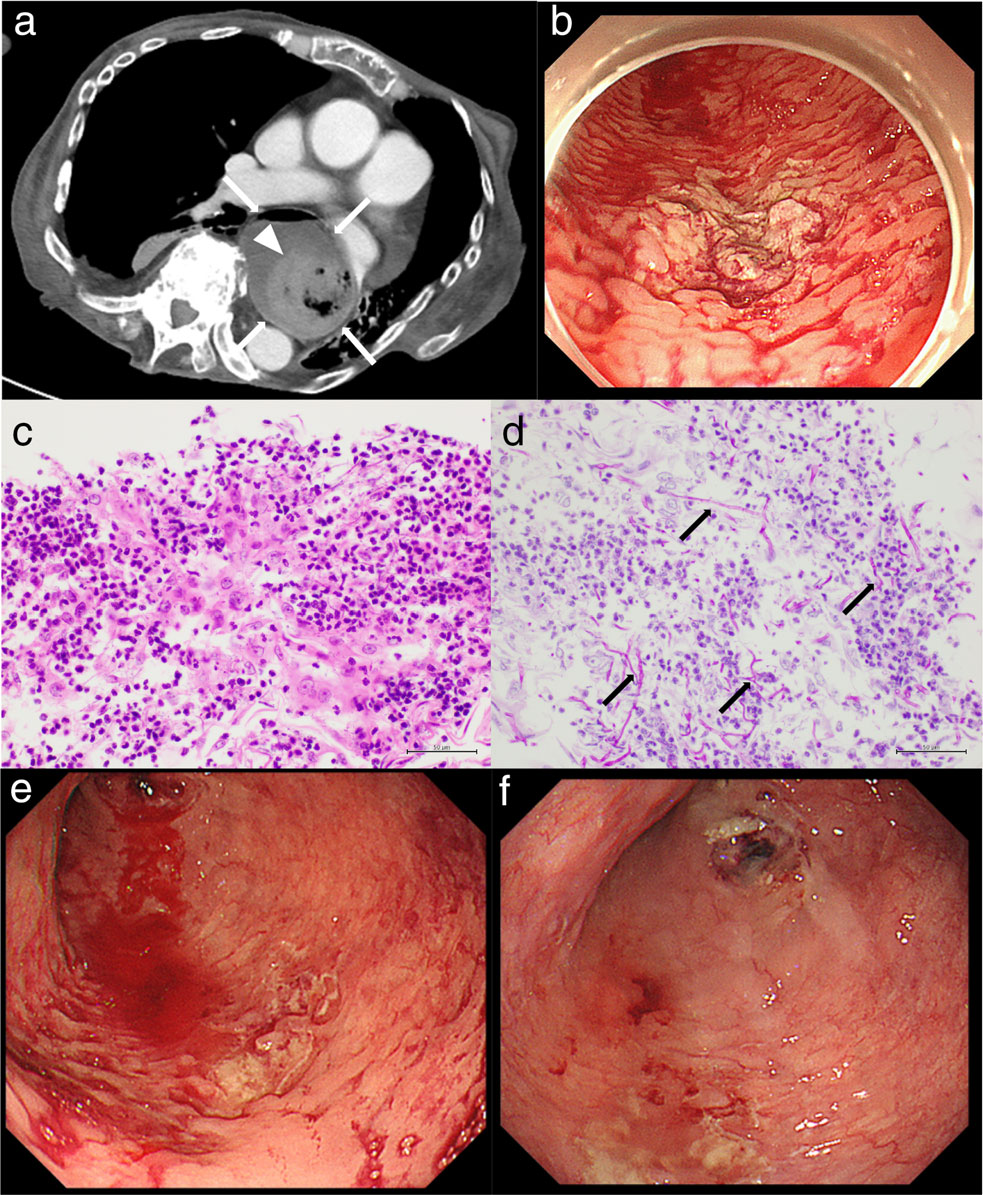
Computed tomography (CT), esophagogastroduodenoscopy (EGD), and pathologic images. (a) CT shows an extremely dilated esophagus (white arrows) with liquid and blood components (white arrowhead). (b) EGD shows multiple large and small ulcers with white plaques, with extravasation and oozing in the upper posterior wall lesion. (c, d) Hematoxylin and eosin and periodic acid–Schiff staining show the severe destruction of the esophageal mucosa with lymphocyte invasion in which infiltrated mycelium (black arrows) are seen. (e) EGD shows reextravasation and oozing of the esophageal ulcers. (f) EGD shows improved esophageal ulcers after the therapies.

A 64‐year‐old woman was diagnosed with rheumatoid arthritis (RA) and SSc 24 and 18 years ago, respectively. She was admitted to our hospital with chief complaints of uncontrolled bleeding from esophageal ulcers and an inability to consume meals. For the RA and SSc, she was being treated with 12 mg of prednisolone daily and 125 mg of abatacept once a week 4 months before presenting to our institution. In addition, she was taking 20 mg of vonoprazan daily as prophylaxis for steroid‐induced gastric ulcers. Approximately 2 weeks ago, she was admitted to the previous hospital for hematemesis. Laboratory examinations from the previous hospital revealed anemia and malnutrition, with the following results: albumin, 2.3 g/dL (normal range: 4.1–5.1 g/dL) and hemoglobin, 7.5 mg/dL (normal range: 11.6–14.8 mg/dL). Computed tomography revealed an extremely dilated esophagus with liquid and blood components (Fig. 1a). Esophagogastroduodenoscopy revealed multiple large and small ulcers with white plaques, with extravasation and oozing seen in the upper posterior wall lesion. The surrounding mucosa was edematous and showed a cobblestone appearance (Fig. 1b). To diagnose the cause of these esophageal ulcers, a biopsy of the ulcer lesions was performed. Hematoxylin and eosin and periodic acid–Schiff staining revealed severe destruction of the esophageal mucosa with lymphocyte invasion, in which infiltrated mycelium was detected (Fig. 1c,d). Based on these results, the esophageal ulcers were considered to be associated with Candida esophagitis (Kodsi classification grade III). The extravasation was treated with cauterization; however, reextravasation and oozing continued, and we repeated the cauterization (Fig. 1e). Daily oral administration of 200 mg of miconazole and 300 mg of amphotericin B was followed by the intravenous administration of 50 mg of micafungin. In addition, we discontinued abatacept, and total parenteral nutrition was initiated along with antifungal therapy soon after admission to our hospital. With these treatments, improvement in esophageal ulcers was observed (Fig. 1f).

## Discussion

A previous study reported that the prevalence of esophageal ulcers caused by Candida esophagitis alone was 2.8%. This was confirmed by excluding other factors such as cytomegalovirus, herpes simplex virus, and gastroesophageal reflux disease.^7^ In our case, SSc may have caused the disturbance in the motility of the esophagus, and the endothelial cell injury may have caused the observed insufficient blood supply.^1^ In addition, the use of immunosuppressive agents (prednisolone and abatacept), acid suppression therapy, and malnutrition were detected. Based on this case, it is important to consider the occurrence of severe Candida esophagitis in patients with an immunosuppressive compromised host and esophageal movement disorders such as SSc. Regular follow up by endoscopy and prophylactic treatment to prevent severe esophagitis may be necessary.

## Declaration of conflict of interest

The authors declare no conflict of interest.
